# Atmospheric Cold Plasma Degradation of Fenvalerate Residues on Shiitake Mushrooms: Mechanisms, Toxicity Evolution, and Quality Effects

**DOI:** 10.3390/foods15071229

**Published:** 2026-04-03

**Authors:** Hu Shi, Ziwen Cheng, Shiwei Dong, Yang Jiao, Hongru Liu

**Affiliations:** 1 Department of Food Science and Technology, Shanghai Ocean University, Shanghai 201306, China; czwzb135@163.com (Z.C.);; 2 Crop Breeding & Cultivation Research Institute, Shanghai Academy of Agricultural Sciences, Shanghai 201403, China

**Keywords:** fenvalerate, pyrethroid, cold plasma, ECOSAR, Fukui functions

## Abstract

Fenvalerate residues on edible mushrooms pose significant risks to food safety and aquatic ecosystems. This study investigated the efficiency, degradation mechanisms, toxicity evolution, and quality effects of atmospheric cold plasma (ACP) for removing fenvalerate from shiitake mushrooms. Fenvalerate degradation increased with ACP treatment voltage and exposure time, reaching a maximum efficiency of 82.5% at 80 kV for 15 min. Quantum chemical calculations based on Fukui functions and frontier molecular orbitals identified phenoxy and chlorophenyl moieties as primary reactive sites. High-performance liquid chromatography–tandem mass spectrometry revealed degradation pathways dominated by hydroxylation, ester bond cleavage, and oxidative transformations. Toxicity assessment using ECOSAR predictions and yeast bioassays demonstrated substantial reductions in acute and chronic toxicity by ACP treatment, although some intermediates retained residual toxicity. In addition, ACP preserved mushroom quality during refrigerated storage. Overall, ACP represents a promising non-thermal strategy for pesticide detoxification while preserving edible mushroom quality.

## 1. Introduction

As the second most cultivated edible mushroom worldwide, shiitake mushrooms (*Lentinus edodes*) are valued for their nutrients such as polysaccharides, proteins, and trace elements [1]. However, shiitake mushrooms cultivation is challenged by pests and diseases, prompting growers to apply broad-spectrum and stable pyrethroid pesticides such as fenvalerate [2]. A survey of 354 mushroom samples from local markets in China revealed that 12.4% contained fenvalerate residues, with concentrations ranging from <0.01 to 0.1 mg/kg [3]. Consequently, fenvalerate residue accumulation has become a major food safety concern in mushrooms, underscored by stringent regulatory limits—as low as 0.01 mg/kg in the European Union [4]. Fenvalerate’s structural stability hinders natural degradation and increases health risks from prolonged low-dose exposure, including effects on human nervous and immune systems [5]. Its toxicity and mobility in the environment further intensify ecological risks especially to aquatic organisms [6,7]. Therefore, effective and safe residue degradation strategies are needed to ensure shiitake mushroom safety and support sustainable industry development [8].

Conventional pesticide-removal methods, such as water washing and thermal treatments, are limited by low efficacy and potential nutrient loss. Water washing is particularly ineffective for hydrophobic pesticides like fenvalerate. For instance, only 19.8% of the similar pyrethroid cypermethrin was removed after 5 min rinsing [9]. Although heating can degrade pyrethroids, it often compromises the nutritional and sensory qualities of food [10]. Emerging technologies such as pulsed electric fields (PEFs) [11], irradiation [12], high-pressure processing (HPP) [13], and ultrasonication [14] have been explored for pesticide removal; however, their practical application is constrained by limitations including high capital cost and non-uniform treatment (PEFs), matrix-dependent efficiency (irradiation), structural damage and high operational cost (HPP), and strong dependence on process parameters (ultrasonication) [15]. In contrast, cold plasma offers a non-thermal, residue-free approach that generates diverse reactive species capable of efficiently degrading pesticides without compromising food quality [16,17]. Numerous studies have demonstrated the potential of ACP for pesticide removal across various fresh commodities such as strawberries, cucumbers, lettuce, maize, tomatoes, and grapes [12,18,19]. Most of these applications have focused on major pesticide classes, particularly organophosphorus insecticides (e.g., chlorpyrifos, diazinon, and phoxim) and common fungicides (e.g., azoxystrobin, fludioxonil, and chlorothalonil) [12,20,21]. Previous studies have demonstrated the effectiveness of cold plasma for pesticide degradation on various substrates. Using plasma-activated water (PAW) combined with sodium molybdate, organophosphorus pesticides were efficiently degraded, achieving a degradation rate of 98.2% [22]. Cold plasma treatment of apples and cucumbers significantly reduced organophosphorus pesticide residues and converted them into less toxic metabolites [19]. A limited number of studies have examined pyrethroid insecticides such as cypermethrin, reporting substantial degradation in mango and tangerine following ACP treatment [23,24], though the underlying mechanisms were not investigated. Despite these advances, research on pesticide degradation in mushrooms remains scarce, particularly for pyrethroids like fenvalerate, and the mechanistic pathways and toxicity of CP-generated transformation products require further elucidation.

This study aims to systematically evaluate the efficacy and underlying mechanisms of atmospheric cold plasma (ACP) in degrading fenvalerate residues on shiitake mushrooms. The objectives are to (1) optimize degradation efficiency by assessing key ACP parameters (voltage and treatment time); (2) elucidate degradation pathways by identifying reactive sites through quantum mechanical calculations and characterizing intermediates using HPLC–MS/MS; (3) assess toxicity of degradation products via ECOSAR modeling and yeast toxicity assays; and (4) examine the effects of ACP treatment on mushroom storage quality.

## 2. Material and Methods

### 2.1. Materials and Chemical Reagents

Around 1 kg of fresh shiitake mushrooms (*Lentinus edodes* around 88.5% moisture content) were purchased from a local Lotus supermarket (Lingang Store, Shanghai, China) with uniform size and absence of visible defects. The mushrooms were stored at 4 ± 1 °C in a refrigerator and all experiments were conducted within 48 h of purchase to maintain freshness. Fenvalerate standard solution (100 mg/L) in acetonitrile (purity ≥ 99%) was obtained from Tianjin Alta Scientific Co., Ltd. (Tianjin, China), and HPLC-grade acetonitrile was supplied by Merck KGaA, Darmstadt, Hesse, Germany. Unless otherwise specified, all other reagents including methanol, purified water, sodium chloride, and anhydrous magnesium sulfate were of analytical grade and sourced from Shanghai Aladdin Biochemical Technology Co., Ltd. (Shanghai, China). Fenvalerate-contaminated mushrooms were prepared by spot spiking 100 μL of a 50 mg/L fenvalerate working solution onto 10 ± 0.1 g mushroom surface, followed by a 30 min equilibration in a fume hood to facilitate penetration, resulting in a final residue of 0.5 mg/kg.

### 2.2. Treating Fenvalerate Contaminated Mushroom with ACP

**Figure 1:**
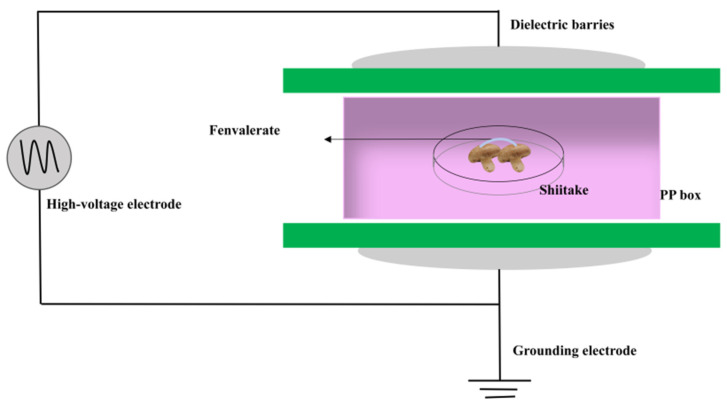
Schematic of atmospheric cold plasma (ACP) treatment of fenvalerate-contaminated shiitake mushrooms.

A dielectric barrier discharge (DBD) cold plasma system was used for treating fenvalerate-contaminated mushroom samples, with the same configuration as in our previous study [25]. As shown in Figure 1, the system consists of a high-voltage power supply (0–100 kV), two circular aluminum plate electrodes, and two polypropylene dielectric layers (2 mm thick). A polypropylene (PP) box (274 × 182 × 42 mm) was placed between the dielectric layers and sealed with a 0.16 mm PET vacuum bag to contain plasma-generated reactive species [26]. Mushroom samples were put on a glass Petri dish at the center of PP box and sealed for treatment. The surface temperature was monitored using an infrared thermometer (model FLIR C5, Teledyne FLIR, Wilsonville, OR, USA) and kept below 40 °C to prevent thermal effects. Plasma parameters were systematically varied—voltage (60, 70, and 80 kV) and duration (0, 2, 5, 10, and 15 min) using an AC power supply operating at 50 Hz, with an estimated discharge power ranging from 75 to 165 W; samples were then analyzed for residual fenvalerate concentrations.

### 2.3. HPLC-MS Analysis of Fenvalerate in Mushroom Samples

Fenvalerate residues on mushroom samples were extracted using a modified QuEChERS method [27]. Briefly, 5 g of homogenized shiitake mushroom sample and 10 mL acetonitrile were added into a 50 mL centrifuge tube and vortexed for 1 min. Then, 4 g anhydrous MgSO_4_ and 1 g NaCl were added, followed by vigorous shaking for 1 min and centrifugation at 8000× *g* rpm for 5 min (model Rotina 380 Hettich Group, Kirchlengern, Germany). For clean-up, 1 mL of the supernatant was transferred to a dSPE tube containing 150 mg anhydrous MgSO_4_ and 50 mg C18 sorbent, vortexed for 1 min, and centrifuged at 10,000 rpm for 3 min. The final extract was filtered through a 0.22 μm nylon syringe filter into an HPLC vial for analysis. HPLC–MS analysis of fenvalerate concentrations was conducted using an Agilent (Santa Clara, CA, USA). ZORBAX SB-C18 column (2.1 × 100 mm, 1.8 μm) maintained at 30 °C. The mobile phase consisted of 0.1% formic acid in water (A) and acetonitrile (B), operated under the following gradient: 0–2 min, 10% B; 2–5 min, 10–90% B; 5–7 min, 90% B; 7–8 min, 90–10% B; and 8–10 min, 10% B. The flow rate was 0.3 mL/min, and the injection volume was 5 μL. The degradation rate of fenvalerates in mushrooms was calculated as follows:

Degradation rate (%) = [(C_0_ − C)/C_0_] × 100,

C_0_ and C represent fenvalerate concentrations in mushrooms (wet basis), where C_0_ and C are the initial and post-treatment concentrations, respectively.

### 2.4. HPLC-MS/MS Analysis for Fenvalerate Degradation Products by ACP

A 50 mg/L fenvalerate solution (1 mL) was spread onto a plastic Petri dish and air-dried at room temperature to near dryness. The dish was then placed in a polypropylene (PP) container, sealed with a polyethylene terephthalate (PET) bag for ACP treatment at 80 kV for 10 min, followed by a 30 min post-treatment time. The residue was reconstituted in 1 mL acetonitrile and filtered through a 0.22 µm PTFE syringe filter prior to analysis. Degradation products were analyzed using a high-resolution HPLC–MS/MS system (Q-Exactive, Thermo Fisher Scientific, Waltham, MA, USA) equipped with a C18 analytical column under the specified experimental conditions. The experimental parameters such as mobile phase, flow rate and injection volume follow above described analytical conditions. Mass spectrometric detection was conducted using an electrospray ionization (ESI) source operated in positive ion mode (ESI+). Data acquisition involved both full scan (*m*/*z* 50–500) and product ion scan modes to identify molecular masses and fragmentation pathways. The transition for fenvalerate was monitored in MRM mode using the precursor ion *m*/*z* 416.1 and product ions *m*/*z* 181.0 and 152.0, with collision energies of 20 eV and 30 eV. The method achieved a detection limit (LOD) of 0.01 mg/kg, a quantification limit (LOQ) of 0.03 mg/kg, and a linear range of 0.01–10 mg/L (R^2^ > 0.999).

### 2.5. Quantam-Mechanic Prediction of Reactive Sites of Fenvalerate

Density functional theory (DFT) calculations were conducted to predict the reactive sites in fenvalerate and elucidate their plasma-induced degradation mechanisms. The molecular structure of fenvalerate was first constructed using GaussView 6.0 and subsequently optimized in Gaussian 16 (Gaussian, Inc., Wallingford, CT, USA). Geometry optimization and vibrational frequency analysis (Opt + Freq) were performed at the B3LYP/6-31G(d,p) level with GD3 dispersion correction to ensure the stability of the optimized structure. This level of theory was chosen for its proven reliability and computational efficiency in modeling organic molecules. Condensed Fukui functions (f^−^, f^+^, and f^0^) were computed using Multiwfn based on Hirshfeld charges to identify atomic sites susceptible to electrophilic (f^−^), nucleophilic (f^+^), and radical (f^0^) attacks. Fukui isosurface maps were generated in GaussView 6.0 to visualize spatial reactivity patterns. By correlating the predicted reactive centers with the structural identities of degradation products detected by HPLC–MS/MS, plausible degradation pathways—such as hydroxylation, ester and cyano bond cleavage, and oxidative transformation—were proposed. Frontier molecular orbital (FMO) analysis was carried out to determine the highest occupied molecular orbital (HOMO) and lowest unoccupied molecular orbital (LUMO), which reflect the electron-donating and electron-accepting capacities of the molecule, respectively. HOMO–LUMO distributions and energy gaps were visualized using GaussView 6.0 by generating orbital isosurfaces (Results → Surfaces/Contours → Create New Surface → Orbital; selecting orbital N for HOMO and N + 1 for LUMO). These descriptors provide insight into the likelihood of electron transfer interactions with reactive species such as ^•^OH and O_3_.

### 2.6. Evaluating Toxicity of Fenvalerate Degradation Products Using ECOSAR and Yeast Assay

The acute and chronic toxicities of fenvalerate and its degradation products were predicted for the following three representative aquatic organisms: fish, water flea, and green algae. Toxicity assessment was performed using the Ecological Structure Activity Relationships (ECOSAR V2.0) software, which applies QSAR models based on molecular structure. Acute toxicity was estimated using LC50 values, representing the concentration of fenvalerate or its degradation products that cause 50% mortality or effect in the tested organisms over standard exposure periods. Chronic toxicity values (ChVs) were also predicted using ECOSAR for all three aquatic species. Toxicity levels were classified according to EPA criteria as follows: highly toxic (<1 mg/L), moderately toxic (1–100 mg/L), and low toxicity (>100 mg/L).

The cytotoxicity of fenvalerate and its ACP-treated degradation products was evaluated using a yeast (Saccharomyces cerevisiae) model, following the method described by [27]. Yeast cells were cultured to the logarithmic phase (OD600 ≈ 0.6); then, aliquots (1 mL) of the yeast suspension were mixed with fenvalerate solutions at specified concentrations and incubated at 30 °C with shaking at 150 rpm for 24 h. After incubation, yeast survival rates were determined. A medium-only group was used as the negative control for comparison. The yeast toxicity assay was conducted in two stages. First, the dose–response relationship between yeast viability and fenvalerate concentration was evaluated using standard solutions of 0, 5, 10, 20, 30, 40, and 50 mg/L. Second, the effect of ACP treatment on the toxicity of fenvalerate was assessed. Fenvalerate solutions (0, 5, 10, 20, and 30 mg/L) were treated with ACP at 80 kV for 10 min, with untreated solutions serving as controls. All experiments were performed in triplicate, and data are presented as mean ± standard deviation.

### 2.7. Quality Evaluation of Mushroom During Storage Following ACP Treatments

During storage, mushroom samples (50 g) were packed in breathable polyethylene bags and stored at 4 °C and 85% relative humidity for 15 days. Quality attributes—including soluble solid content, total phenols, and texture (hardness)—were evaluated at three-day intervals. Texture analysis was performed using a texture analyzer (TA.XT PlusC, Stable Micro Systems Ltd., London, UK) equipped with a P/5 probe (5 mm diameter), following established procedures. The test conditions were: pre-test speed 3 mm/s, test speed 1 mm/s, post-test speed 3 mm/s, test depth 10 mm, and trigger force 10 g. Hardness values represent the mean of ten measurements per treatment group. Total phenolic content was determined using the Folin–Ciocalteu method [28]. Mushroom samples were extracted with 70% ethanol by ultrasonication, followed by centrifugation to obtain the supernatant. The reaction mixture was prepared by adding Folin–Ciocalteu reagent and sodium carbonate solution to the extract, allowing color development at room temperature. Absorbance was measured at 760 nm, and total phenols were calculated from the calibration curve. Total soluble solids (TSSs) were measured using a handheld digital refractometer (model LC-DR-53B, Shanghai Lichen Tech Corp, Shanghai, China). After clarification of the ethanol extract, 1–2 drops were placed on the prism surface, and the stabilized reading was recorded. Each sample was measured in triplicate, and results are expressed as mean ± standard deviation.

### 2.8. Data Processing and Illustration

Data processing and statistical analysis were performed using SPSS 25.0 (IBM Corp, Armonk, NY, USA). All experimental values are presented as mean ± standard deviation (SD). Single-factor analysis of variance (ANOVA) was used, and significant differences between means were determined using Duncan’s multiple range test at *p* < 0.05. Except for texture analysis, all experiments were performed in triplicate to ensure statistical reliability. ChemDraw 19.0 (CambridgeSoft Corp, Cambridge, MA, USA) was used to illustrate compound formulas and degradation pathways.

## 3. Results and Discussion

### 3.1. Effect on Fenvalerate Degradation on Mushroom by Cold Plasma

**Figure 2:**
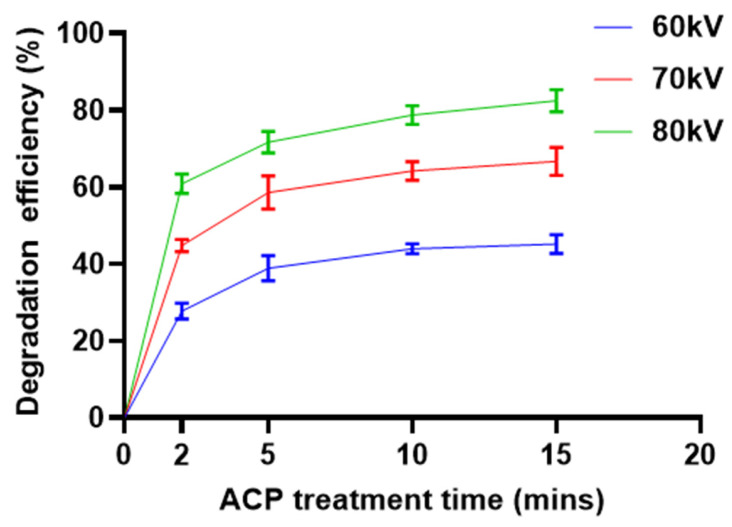
Effects of ACP voltage and treatment time on fenvalerate degradation on shiitake mushroom surfaces.

The degradation efficiency (%) of fenvalerate on mushroom surfaces under atmospheric cold plasma (ACP) at varying voltages and treatment durations is shown in Figure 2. Fenvalerate degradation efficiency increased with both voltage and exposure time, reaching a maximum of 82.5% at 80 kV with 15 min of treatment. Most degradation occurred within the first 2–5 min, underscoring the rapid reactivity of ACP-generated species with fenvalerate residues. At lower voltages (60 and 70 kV), the highest observed degradation efficiencies were 45.2% and 66.6%, respectively. These results are comparable to previous reports on other pyrethroid pesticides. For example, a 5 min gliding arc discharge treatment reduced cypermethrin residues in mango by 62.9% [23] and nonthermal plasma processing achieved a 75% reduction in cypermethrin in tangerine [24]. Pesticides degradation by cold plasma is primarily attributed to reactive oxygen and nitrogen species (RONS) generated during ACP exposure—such as O_3_, ^•^OH, H_2_O_2_, NxOy—which promote oxidative reactions including hydroxylation and bond cleavage [29]. In addition to ^•^OH radicals, other reactive species generated during ACP, such as ozone (O_3_), nitrogen oxides (NOx), high-energy electrons, and UV photons, may also contribute to pesticide degradation. The strong positive correlation between voltage and degradation efficiency can be explained by the increased electron energy at higher discharge voltages, which enhances gas excitation and ionization, thereby producing more reactive species [18,25].

### 3.2. Prediction of Reactive Sites Based on Quantum Chemical Calculations

**Figure 3:**
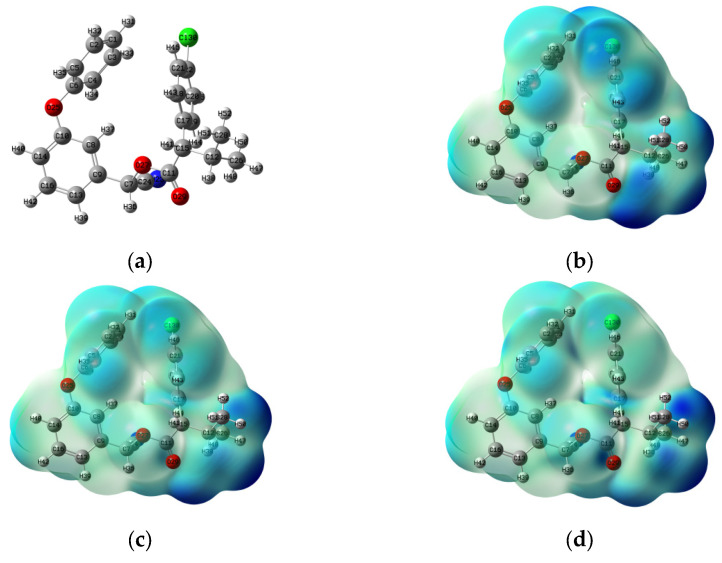

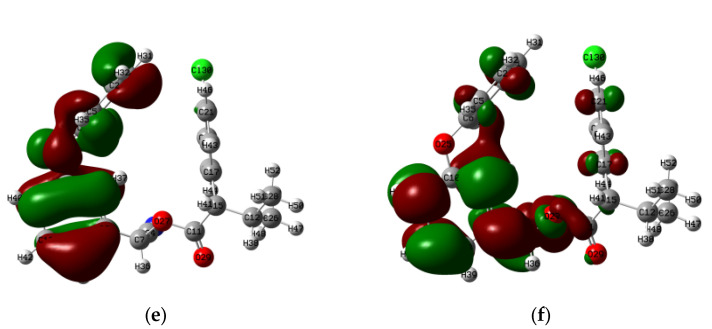
Quantum chemical analysis of fenvalerate: (**a**) molecular structure; condensed Fukui functions for (**b**) electrophilic (f^−^), (**c**) radical (f^0^), and (**d**) nucleophilic (f^+^) attack; (**e**) HOMO and (**f**) LUMOs.

The Fukui function describes the spatial distribution of a molecule’s reactivity toward electron gain or loss, enabling the identification of regions susceptible to electrophilic, nucleophilic, or radical attack [30]. While frontier molecular orbitals (HOMO and LUMO) largely dictate a molecule’s electron-donating and electron-accepting behavior [31], the condensed Fukui function (CFF) provides a quantitative approach to evaluate the reactivity of individual atoms toward nucleophilic (f^+^), electrophilic (f^−^), and radical (f^0^) reactions [32]. f^+^ indicates sites susceptible to nucleophilic attack, f^−^ indicates sites susceptible to electrophilic attack, and f^0^ indicates susceptibility to radical attack. This classification helps to interpret the reactivity patterns of the molecule. For fenvalerate, the molecular structure and Fukui function isosurfaces are shown in Figure 3a–d. Atoms Cl-52, C-12, O-26, C-36, C-39, and C-7 exhibit high f^−^ values and are tightly enclosed by the f^−^ iso-surface, indicating strong susceptibility to electrophilic attack. Similarly, Cl-52, C-7, C-12, O-26, and C-36 show elevated f^0^ values, suggesting increased radical reactivity. Thus, the phenoxy group (C-12, O-26, and C-7) and chlorophenyl group (Cl-52, C-36, and C-39) are predicted as the most reactive regions. The HOMO and LUMO distributions of fenvalerate (Figure 3e,f) further support these predictions. The LUMO is primarily located on the phenoxy-phenyl ring and atoms N-15, C-10, C-6, and O-4, identifying these positions as the most favorable sites for nucleophilic attack. In contrast, the HOMO is concentrated on the phenoxy ring, with significant contributions from the chlorophenyl moiety, O-24, and C-10, indicating that these atoms are prone to electrophilic attack and electron loss [6]. The consistency between Fukui function analysis and frontier orbital theory reinforces the reliability of the predicted reactive sites. Both methods point to the phenoxy and chlorophenyl groups as the major sites of electrophilic attack (high f^−^ and HOMO density). For nucleophilic reactivity, the LUMO highlights the ester bond and cyano group as the most probable sites for hydrolysis, whereas f^0^ identifies additional potential reactivity within the aromatic rings. These two approaches thus provide complementary insights into fenvalerate’s degradation behavior. Notably, the high reactivity of Cl-52 is consistent with the polarity of the C–Cl bond, making it vulnerable to high-energy electrons or ^•^OH radicals, leading to dechlorination or hydroxylation reactions. The LUMO distribution on the phenoxy group further suggests that this moiety may act as an electron acceptor during oxidative processes, contributing to ester bond cleavage or aromatic ring opening [33,34].

### 3.3. Degradation Products and Pathways of Fenvalerate by ACP Treatments

**Figure 4:**
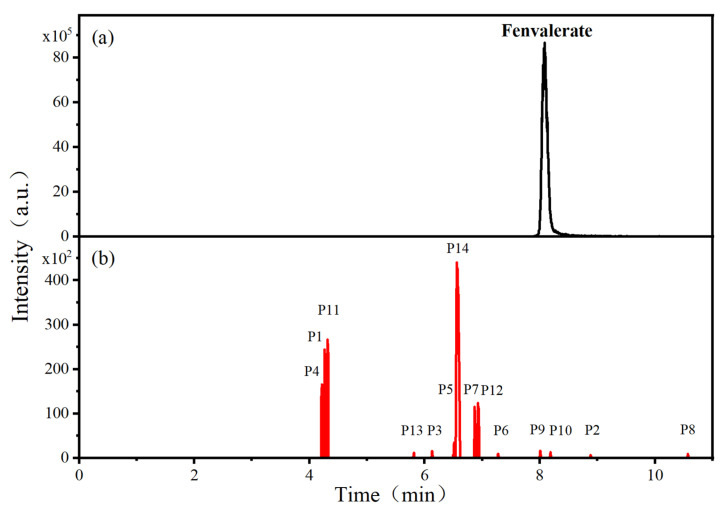
HPLC–MS/MS total ion chromatograms of fenvalerate: (**a**) untreated sample and (**b**) ACP-treated sample.

**Figure 5:**
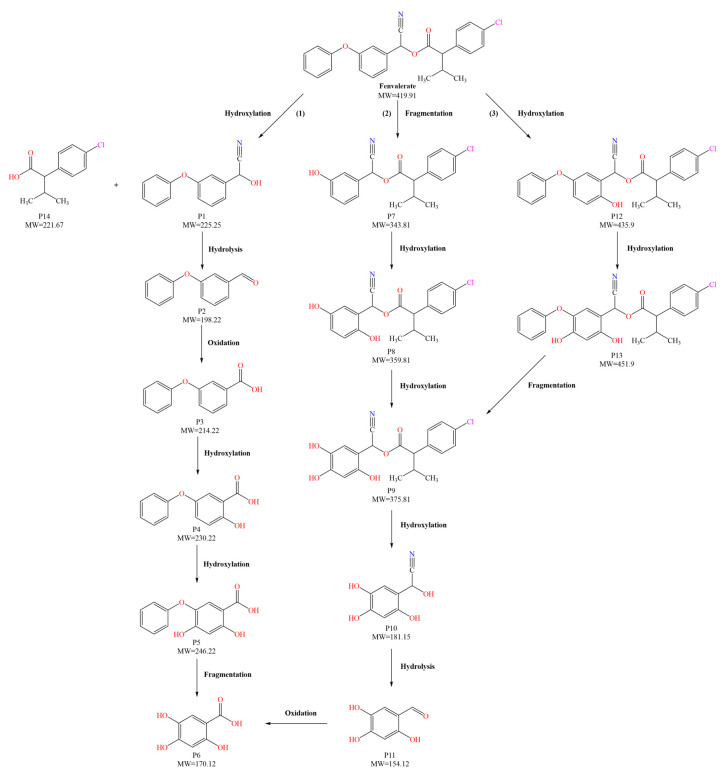
Proposed degradation pathways of fenvalerate during ACP treatment.

Figure 4 presents the total ion chromatograms of untreated and plasma-treated fenvalerate samples. In the untreated sample, a prominent peak at 8.08 min (*m*/*z* 419.13) corresponded to fenvalerate, whereas this peak diminished following ACP treatment, accompanied by the appearance of 14 new peaks representing degradation products. The molecular formula and structure of fenvalerate degradation products were characterized using Xcalibur software (V4.3) [33]. The molecular formula, retention times, and proposed molecular structures of all identified compounds are summarized in Table 1. Several degradation products identified in this study are consistent with those previously reported in the fungal biodegradation of fenvalerate. Notably, intermediates such as 3-phenoxybenzaldehyde (P2), 3-phenoxybenzoic acid (P3), and 2-(4-chlorophenyl)-3-methylbutanoic acid (P14) were also observed in [35]. Based on HPLC-MS/MS results and DFT-predicted reactive sites, three primary degradation pathways were proposed as illustrated in Figure 5.

**Table 1:** Proposed formulas and structure for degradation products of fenvalerate by atmospheric cold plasma treatment based on HPLC−MS/MS analysis.

Compound	Structure	Molecular Formula	Retention Time (min)	Actual Mass (*m*/*z*)	Measured Mass (*m*/*z*)	Mass Error (*m*/*z*)
Fenvalerate	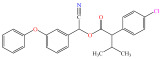	C_25_H_22_ClNO_3_	8.08	419.13	419.13	All numbers below modified in clean version
P1	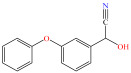	C_14_H_11_NO_2_	4.27	225.08	225.08	0.00
P2	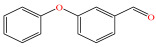	C_13_H_10_O_2_	8.88	198.07	198.04	0.03
P3	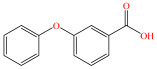	C_13_H_10_O_3_	6.13	214.06	214.08	0.02
P4	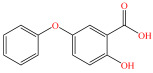	C_13_H_10_O_4_	4.21	230.06	230.01	0.05
P5	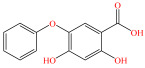	C_13_H_10_O_5_	6.52	246.05	246.07	0.02
P6	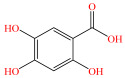	C_7_H_6_O_5_	7.28	170.02	170.06	0.04
P7	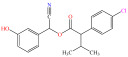	C_19_H_18_ClNO_3_	6.86	343.10	343.29	0.19
P8	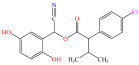	C_19_H_18_ClNO_4_	10.57	359.09	359.22	0.13
P9	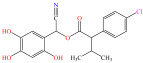	C_19_H_18_ClNO_5_	8.01	375.09	375.28	0.19
P10	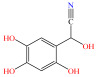	C_8_H_7_NO_4_	8.19	181.04	181.03	0.01
P11	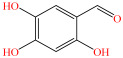	C_7_H_6_O_4_	4.32	154.03	154	0.03
P12	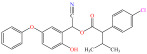	C_25_H_22_ClNO_4_	6.92	435.12	435.12	0.00
P13	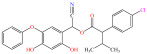	C_25_H_22_ClNO_5_	5.81	451.12	451.07	0.05
P14	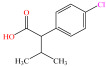	C_11_H_13_ClO_2_	6.55	212.06	212.06	0.00

1. The first pathway initiates with ^•^OH radical attack at the C29–O4 bond of fenvalerate, producing 2-hydroxy-2-(3-phenoxyphenyl)acetonitrile (P1) and 2-(4-chlorophenyl)-3-methylbutanoic acid (P14). P1 undergoes hydrolysis to form 3-phenoxybenzaldehyde (P2), which is further oxidized to 3-phenoxybenzoic acid (P3). Guided by DFT predictions, successive ^•^OH attacks at C12 and C7 positions of P3 generate hydroxylated intermediates (P4 and P5). Subsequent cleavage of the O26–C27 bond in P5 yields 2,4,5-trihydroxybenzoic acid (P6). This sequence is consistent with the ^•^OH-mediated degradation mechanism reported by [36].
2. The second pathway begins with cleavage of the O26–C27 bond in fenvalerate, forming intermediate P7. Hydroxylation at C12 and C7 leads to P8 and P9, respectively. Further ^•^OH attack at the C29–O4 bond of P9 produces P10, which hydrolyzes to P11 and subsequently oxidizes to P6.
3. In the third pathway, fenvalerate undergoes sequential hydroxylation at C12 and C7 to form intermediates P12 and P13. Cleavage of the O26–C27 bond in P13 generates P9, linking this route to the later stages of Pathway 2. Overall, these results suggest that ^•^OH radicals play a dominant role in fenvalerate degradation during ACP treatment, promoting hydroxylation, oxidation, and ester bond cleavage to yield progressively smaller and more polar intermediates.

### 3.4. ECOSAR Prediction of Fenvalerate Degradation Product Toxicity

**Figure 6:**
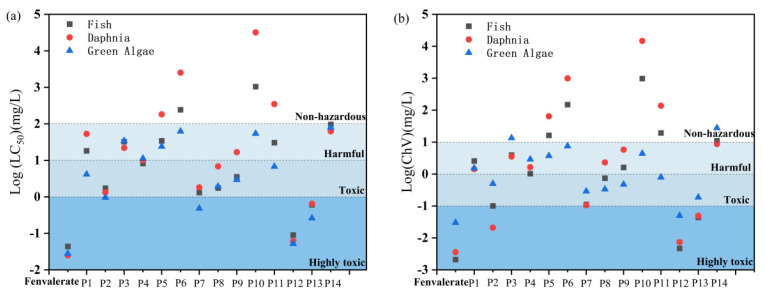
ECOSAR-predicted (**a**) acute and (**b**) chronic toxicity of fenvalerate and its ACP degradation products.

The acute and chronic toxicities of fenvalerate and its degradation products to fish, daphnia, and green algae were predicted using the ECOSAR software (V2.0), as summarized in Figure 6. Fenvalerate exhibited high toxicity toward all three aquatic species, with acute LC50 values of 0.044, 0.025, and 0.028 mg/L, and chronic ChV values of 0.0021, 0.0036, and 0.030 mg/L, respectively. After ACP treatment, most degradation products showed substantially reduced both the acute and chronic ecotoxicity than fenvalerate. Major intermediates such as P5, P6, and P10 were non-toxic to fish and Daphnia magna. However, P12 remained highly toxic to all tested organisms, and P2, P7, and P11 exhibited moderate toxicity toward green algae. Persistence of certain toxic intermediates (e.g., P12) highlights the need for targeted risk assessment of specific degradation products. Further optimization of plasma parameters, particularly increasing power intensity, may enhance degradation efficiency and facilitate the conversion of toxic intermediates into less harmful or non-toxic small molecules. Similar trends have been reported in other plasma degradation studies, where non-thermal plasma treatments markedly reduced the overall toxicity of organic pollutants such as sulfamethoxazole [35].

### 3.5. Toxicity of Degradation Product of Fenvalerate Using Yeast Assay

**Figure 7:**
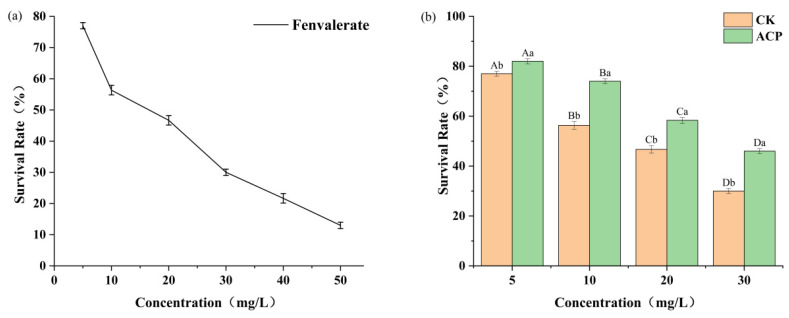
Effects of fenvalerate concentrations (**a**) and ACP treatment (**b**) on yeast survival rate.

**Figure 8:**
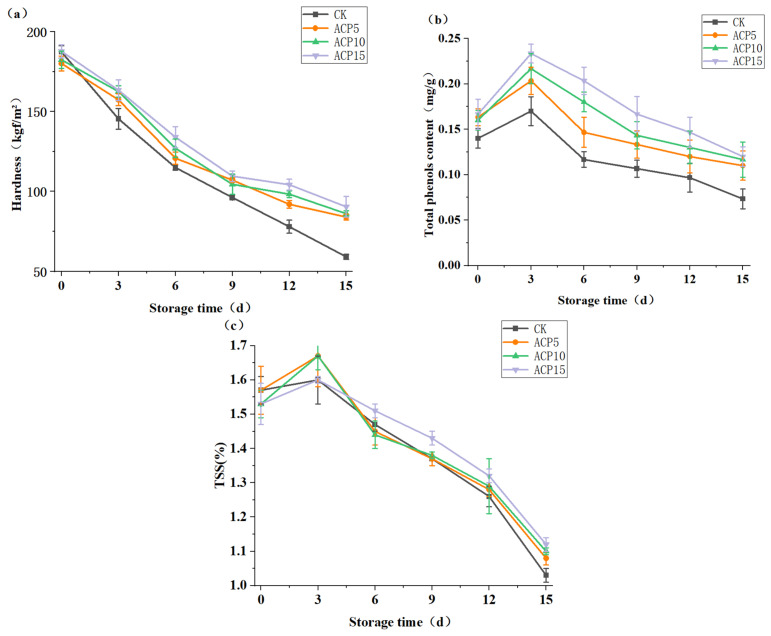
Effects of ACP treatment on the quality attributes of shiitake mushrooms during storage. (**a**) Hardness, (**b**) total phenols (TPs), (**c**) total soluble solids (TSSs).

Figure 7 illustrates the relationship between fenvalerate concentration and yeast survival. A clear inverse correlation was observed, with increasing fenvalerate concentrations exerting progressively stronger inhibitory effects on yeast viability. The median lethal concentration (LD50) of fenvalerate for yeast (Saccharomyces cerevisiae) was estimated at 16.56 mg/L using probit analysis. The protective effect became more pronounced at higher initial fenvalerate concentrations. The greatest improvement was observed at 30 mg/L, where ACP treatment (80 kV, 10 min) increased yeast survival from 30% to 46%, corresponding to a reduction in fenvalerate concentration from approximately 30 mg/L to 8.7 mg/L (Figure 8a). Based on the concentration–response relationship, the survival rate for pure fenvalerate at 8.7 mg/L would be expected to be approximately 60.2%. This difference may be attributed to the formation of intermediate degradation products during ACP treatment, which may retain residual toxicity or exhibit synergistic effects. In addition, the concentration–response model was established based on the parent compound and may not fully reflect the combined effects of multiple degradation products.

### 3.6. Effects of Cold Plasma Treatment on the Quality of Shiitake Mushrooms

Hardness of shiitake mushrooms declined progressively during storage in all groups (Figure 8a), reflecting the enzymatic breakdown of cell wall components such as cellulose, chitin and glucans. Similar trends have been reported in other produce, where cold plasma slowed tissue softening by inhibiting cell wall–degrading enzymes [37]. In this study, ACP treatment showed the same protective effect: ACP15 group maintained the highest hardness 90.40 g at day 15, compared with 59.08 g in the control. Phenolic compounds act as key antioxidants and browning substrates in shiitake mushrooms, making total phenolics (TPs) an important freshness indicator. During storage, TP followed the typical pattern of an initial rise and subsequent decline (Figure 8b). ACP-treated mushrooms showed slightly higher initial TP (0.17 mg/g) levels than the control (0.14 mg/g). This phenomenon persisted throughout storage: by day 15, ACP-treated mushrooms retained higher TP (0.12 mg/g) than the control (0.07 mg/g). The preservation of TP by ACP treatment may result from inactivation of polyphenol oxidase (PPO) and/or a mild physiological stress response, which stimulate antioxidant metabolism and help maintain phenolic content [38]. Thus, ACP treatment helped slow phenolic degradation and preserve antioxidant capacity. Total soluble solids (TSSs) as an indicator of shiitake mushroom maturity declined during storage in all groups (Figure 8c). No significant differences in TSS were observed between the control and ACP-treated samples, indicating that ACP treatment does not adversely affect the metabolic processes responsible for soluble solids, and overall maturation and sweetness were maintained, consistent with findings reported by [11]. Overall, ACP demonstrated effective quality-preserving effect on shiitake mushrooms by mitigating tissue softening, sustaining phenolic content and antioxidant capacity, and maintaining TSS levels throughout storage.

## 4. Conclusions

This study comprehensively evaluated the degradation efficiency, underlying mechanisms, and quality effects of atmospheric cold plasma (ACP) in removing fenvalerate residues from shiitake mushrooms. Under optimized conditions (80 kV, 15 min), ACP achieved an 82.5% degradation rate. Quantum chemical calculations revealed that the phenoxy and chlorophenyl groups as primary reactive sites, HPLC-MS/MS revealed degradation proceeding through hydroxylation, ester bond cleavage, and oxidative pathways, ultimately yielding small organic acids and phenolic derivatives. Toxicological assessment using ECOSAR prediction indicated markedly reduced acute and chronic toxicity of these degradation products toward fish, daphnia, and green algae, while yeast bioassays further confirmed enhanced biological safety, as evidenced by increased cell viability following ACP treatment. ACP treatment effectively preserved mushroom hardness and extended shelf life by mitigating tissue softening and maintaining phenolic content and total soluble solids during storage. Collectively, these results highlight ACP as a promising approach for pesticide residue control, offering efficient degradation, reduced toxicity, and minimal impact on product quality. This study was conducted at the laboratory scale; industrial application would require further evaluation of energy consumption, processing costs, and quality maintenance. Further refinement of plasma parameters and elucidation of reactive species contributions will support broader application across diverse edible fungi and pesticide types.

## Data Availability

The original contributions presented in this study are included in the article. Further inquiries can be directed to the corresponding authors.
